# Mental health consultations in a prison population: a descriptive study

**DOI:** 10.1186/1471-244X-6-27

**Published:** 2006-06-07

**Authors:** Ellen Kjelsberg, Paal Hartvig, Harald Bowitz, Irene Kuisma, Peder Norbech, Aase-Bente Rustad, Marthe Seem, Tom-Gunnar Vik

**Affiliations:** 1 Centre for Research and Education in Forensic Psychiatry, Ulleval University Hospital, Building 7 Gaustad, NO-0320 Oslo, Norway; 2 Psyk. poliklinikk Baerum, Postboks 83, NO-1309 Rud, Norway; 3 Oslo Prison, P.O.Box 9292, NO-0134 Oslo, Norway; 4 Jessheimklinikken, Trondheimsveien 75, NO-2050 Jessheim, Norway; 5 Ringerike Prison, P.O.Box 40, NO-3533 Tyristrand, Norway; 6 Telemark Hospital, Ulefossveien 55, NO-3710 Skien, Norway

## Abstract

**Background:**

The psychiatric morbidity among prison inmates is substantially higher than in the general population. We do, however, have insufficient knowledge about the extent of psychiatric treatment provided in our prisons. The aim of the present study was to give a comprehensive description of all non-pharmacological interventions provided by the psychiatric health services to a stratified sample of prison inmates.

**Methods:**

Six medium/large prisons (n = 928) representing 1/3 of the Norwegian prison population and with female and preventive detention inmates over-sampled, were investigated cross-sectionally. All non-pharmacological psychiatric interventions, excluding pure correctional programs, were recorded. Those receiving interventions were investigated further and compared to the remaining prison population.

**Results:**

A total of 230 of the 928 inmates (25 %) had some form of psychiatric intervention: 184 (20 %) were in individual psychotherapy, in addition 40 (4 %) received ad hoc interventions during the registration week. Group therapy was infrequent (1 %). The psychotherapies were most often of a supportive (62 %) or behavioural-cognitive (26 %) nature. Dynamic, insight-oriented psychotherapies were infrequent (8 %). Concurrent psychopharmacological treatment was prevalent (52 %). Gender and age did not correlate with psychiatric interventions, whereas prisoner category (remanded, sentenced, or preventive detention) did (p < 0.001). Most inmates had a number of defined problem areas, with substance use, depression, anxiety, and personality disorders most prevalent. Three percent of all inmates were treated for a psychotic disorder. Remand prisoners averaged 14 sessions per week per 100 inmates, while sentenced inmates and those on preventive detention averaged 22 and 25 sessions per week per 100 inmates, respectively. Five out of six psychiatric health services estimated the inmates' psychiatric therapy needs as adequately met, both overall and in the majority of individual cases.

**Conclusion:**

Our results pertain only to prisons with adequate primary and mental health services and effective diversion from prison of individuals with serious mental disorders. Given these important limitations, we do propose that the service estimates found may serve as a rough guideline to the minimum number of sessions a prison's psychiatric health services should be able to fulfil in order to serve the inmates psychiatric needs. The results rely on the specialist services' own estimates only. Future studies should take other important informants, including the inmates themselves, into consideration.

## Background

Even with the most disordered prison inmates diverted from our prison systems, there remain a large number of inmates suffering from a variety of mental disorders [1-5]. Severe personality disorders and substance-use disorders are prevalent. These disorders are often chronic and do not lend themselves easily to therapeutic interventions [6]. Disorders and complaints more amenable to treatment are also prevalent, such as depression, anxiety disorders, post-traumatic stress disorders, and sleep disorders [3,4,7]. However, even these disorders are often co-morbid with deviant personality and/or substance abuse [8-10].

Prison inmates have the right to mental health care equivalent to that available to the rest of the population [11,12]. Even so, studies have shown that few patients receive adequate psychiatric services during imprisonment despite high levels of perceived need [3,13-17]. This might indeed represent a missed opportunity to ameliorate the mental health status of individuals that do not usually come into contact with mental health services.

A number of 'therapeutic' programs, focusing on crime relapse prevention, are described in the literature. They differ greatly, from US boot camps to encounter groups and transactional analysis [18]. The programs are mainly concerned with public safety and have crime reduction as their primary goal. Although we acknowledge the importance of these programs, they are not included here: the present paper concerns itself with psychiatric interventions whose primary goal is to comfort and ease the burden of disease and, if possible, to cure.

So how much psychiatric treatment is currently provided in our prisons? And how much is needed? We have found few naturalistic studies describing psychiatric practices in prison settings [13,19-21] and even fewer studying prison inmates' treatment needs [3,22]. To our knowledge, no-one has described the psychiatric services required in order to serve a prison population adequately.

The aim of the present study was to conduct a naturalistic study of all psychiatric activity in a large prison population. Psychotherapists working in prisons provide a variety of services, from emergency interventions through ad hoc supportive consultations to conventional psychotherapies. We wanted to describe all these activities comprehensively. In addition, we wanted to describe the patients in some detail and compare them with the rest of the prison population, in order to identify factors associated with receiving psychiatric interventions. As we expected systematic differences with regard to gender and prisoner category, females and prisoners on preventive detention were over-sampled.

## Methods

### The Psychiatric Health Services (PHS) in Norwegian prison

The PHS in Norwegian prisons are fully integrated into the country's general health services. They are administratively and economically independent of the Correctional Services and are ultimately the responsibility of the Department of Health and Social Welfare, not the Department of Justice. Hence, all prison health workers are independent of the correctional facility they service, both administratively and financially. There are no forensic psychiatric hospitals in Norway.

Within the first week of incarceration, all new prisoners are screened for possible somatic or psychiatric health problems. This is usually done in a personal interview by a primary health worker. Necessary treatment is offered and referral to specialist services is effectuated when required. Hence, much rests on the discretion of the primary health services.

Most PHSs are cross-disciplinary teams working in close co-operation with the prison officers and the prison's primary health services. Usually, each prison inmate is assigned one particular prison officer as his or her primary contact, equivalent to the primary nurse system in health institutions. If the inmate has mental problems of any kind, the primary prison officer will arrange for a consultation with a primary health care worker. Only if the ensuing primary health care intervention does not produce the desired result or if the problem is of a nature obviously requiring specialist attention, does the primary health service arrange for the inmate to see a PHS therapist.

The present paper reports exclusively on the PHS's clinical work with prison inmates. A number of other important PHS tasks, such as psycho-educative and consultative work with prison officers and the primary health services and follow-up of inmates recently released to the community, are outside the scope of this study.

### The study population

In Norway, remanded and sentenced prisoners are incarcerated together in the same institutions. At the time of the study (May 2005), 2977 persons, 2812 males (94.5 %) and 165 females (5.5 %), were incarcerated in Norway. Of these, 602 (20.2 %) were remanded, 71 (2.4 %) were on preventive detention, and the remaining 2304 (77.4 %) were serving regular sentences.

We did not find it feasible to investigate the country's total prison population (44 prisons). Instead, we selected six medium/large prisons, representing 1/3 of the total prison population. One of the prisons selected was Bredtveit (N = 52), Norway's largest female prison, thereby ensuring that gender differences could be explored. In addition, we chose Ila (N = 107), the prison responsible for most individuals on preventive detention, thus enabling us to investigate this important subgroup. The remaining four prisons (mean number of inmates 192, range 82–360) were chosen because they were considered representative for the country's larger prisons with medium to high security profiles. Hence, our study population is a stratified sample of Norwegian prisons. The selection procedure excludes us from drawing any conclusions as to psychiatric activities in small prisons with low security profiles.

A total of 928 individuals were incarcerated in the six prisons investigated, 865 (93 %) males and 63 (7 %) females. This represented 31 % of the country's total prison population at the time, 31 % of all males and 38 % of all females.

In official prison statistics, ages are collapsed into age groups. The age distribution among the 928 prisoners were as follows: under 21: 46 (5 %), 21–25: 153 (16 %), 26–30: 176 (19 %), 31–40: 331 (36 %), 41–50: 160 (17 %), 51–60: 52 (6 %), and above 60: 10 (1 %). The age distribution did not differ significantly from that of the remaining prison population.

While 265 (29%) were remand prisoners, 602 (65 %) were sentenced inmates, and 61 (7 %) were on preventive detention. Among the 928, 623 (67 %) were Norwegian citizens, the remaining 305 (33 %) were of nationalities representing the continents as follows: the rest of Europe 163 (53 % of all non-Norwegians), Asia 71 (23 %), Africa (18 %), and America (5 %). In addition, one prisoner was stateless.

We did not attempt to measure the prevalence of mental disorders in the population under investigation.

### Questionnaires

A questionnaire, intended to capture the overall functioning of the psychiatric services at each participating prison, was developed. It included questions about such diverse issues as staffing, emergency routines, consultation rooms, and co-operation with other agencies. In addition, the respondent was asked to give a global assessment of whether the psychiatric treatment needs of the total inmate population at that particular prison were adequately met. In their assessments they did also incorporate input from the primary health services. A senior psychotherapist at each of the participating mental health services (this paper's authors 3–8) completed the questionnaire.

An additional questionnaire was developed in order to collect information about each inmate receiving any kind of psychiatric intervention during one specific week in May 2005. All inmates currently in group or individual psychotherapy were included, regardless of whether a session did indeed take place during that specific week or not. In addition, all individuals receiving ad hoc or emergency psychiatric interventions during the registration week were included.

Inmates in therapy with therapists other than those in the PHS were also included. These were infrequent and included, in addition to a few private psychiatrists/psychologists, a clinic for treatment of deviant sexuality. The prisons' primary health services, in particular experienced psychiatric nurses, did undoubtedly provide important mental health services to a number of inmates. However, as the aim of the present study was to describe the activity at the PHS level, this activity was not included in the present material.

The vast majority of patient questionnaires were filled out by the individual psychotherapist and included information about gender, chronological age (as opposed to official prison statistics that uses age categories only), ethnicity, prisoner category, and details about the services provided. The therapist was asked to assess whether the inmate's psychiatric problems were within the realm of one or more of the following categories: psychoactive substance use, psychosis, personality deviance, depression, anxiety, posttraumatic stress, or any other problem (in which case he/she was asked to specify its nature). In addition, the therapist was asked to assess whether that particular patient's treatment needs were adequately met.

The study was approved by the Norwegian National Committee for Medical Research Ethics (Ref. 128–05044).

## Results

### The psychiatric health services

Clerical and administrative staff aside, various professional therapists (psychiatric nurses, psychologists, and psychiatrists) worked in the six PHSs studied, a number of these on a part-time basis. All in all 9 therapist positions were filled, amounting to roughly 1 therapist per 100 inmates. All but one of the participating PHSs were satisfied with the therapeutic capacity, with minimal or no waiting lists in operation.

All PHSs reported that they were adequately serviced by their general psychiatric hospitals with respect to transfer of inmates needing hospitalisation. However, 4 of the 6 PHSs had experienced problems with patients being sent back to prison too soon.

All PHSs reported good relationships with prison authorities, prison officers, and the primary health services and found this collaboration of crucial importance. A number of psychiatric nurses were working in the prisons' primary health services, and their role in securing optimal functioning of the total health service was particularly emphasised.

Many of the therapists drew attention to the problems inherent in doing psychiatric work in a prison setting.

### Activities

Among the 928 inmates, 230 (25 %) received some form of psychiatric intervention. The majority (218 inmates, 95 %) had individual interventions only. Twelve inmates were in group therapy; six of these were in individual therapy as well.

A total of 183 inmates (82 % of the 224 in individual therapy) received some kind of planned psychiatric intervention: 76 had sessions once a week (or more), 106 had sessions 1–3 times a month, and 5 even more infrequently. In addition, 40 inmates received ad hoc or emergency interventions during the registration week.

The estimated duration of therapies varied considerably, from 1 to 48 months, with an average duration of 10 months (SD = 8.4, median 7). The estimated number of sessions varied from 4 to 192 (mean 27, SD = 28, median 20).

The sessions were conducted in various settings, depending on building facilities and necessary security measures. In one prison 90 % of all therapies were conducted in the inmate's cell, in another 100 % in other localities on the premises. Only 6 % of sessions took place outside the prison.

Individual therapy was most often of a supportive nature (62 %). Cognitive-behavioural therapy was also relatively frequent (26 %), while dynamically oriented therapy, focusing on insight, was relatively infrequent (8 %).

The therapist's profession was psychologist in 52 %, psychiatric nurse in 24 %, and psychiatrist in 23 % of the cases. Only 1 % of the therapists were social workers.

### Characteristics of inmates receiving interventions

Demographic details of the study population are given in Table 1. Gender and age did not correlate with being in therapy, whereas prisoner category (remanded, sentenced, or preventive detention) did (Chi square 17.02, 2DF, p < 0.001). Sentenced prisoners were most likely to have a psychiatric intervention, remand prisoners least likely.

**Table 1:** Demographic factors for 928 prison inmates, according to receiving psychiatric interventions or not.

	**Inmates receiving psychiatric interventions N = 230**		**Inmates without psychiatric interventions N = 698**		**Total prison population N = 928**	
	
	n	%	n	%	n	%
**Gender**						
Males	210	24	655	76	865	93
Females	20	32	43	68	63	7
**Age group**						
Under 21	8	17	38	83	46	5
21–25	41	27	112	73	153	17
26–30	50	28	126	72	176	19
31–40	77	23	254	77	331	36
41–50	43	27	117	73	160	17
51–60	10	19	42	81	52	6
Over 60	1	10	9	90	10	1
Prisoner category ***						
Remand prisoner	42	16	223	84	265	29
Sentenced	172	29	430	71	602	65
Preventive detention	16	26	45	74	61	7

*** Chi square test = 16.05, 2DF: p ≤ 0.001

A total of 150 (65 %) of the inmates receiving interventions were ethnic Norwegians. The remaining 80 were of the following ethnic origin: European 14, Asian 35, African 18, South American 6, and non-specified 7. Even so, 92 % of the inmates were fairly fluent in Norwegian. Five percent had some language difficulties and 3 % did not speak Norwegian. However, as English in some cases could be used as a common second language, only 3 inmates were found to be in need of a translator.

The therapists' assessments of their patients' psychiatric problem areas, including gender-specific data, are listed in Table 2. Most inmates (79 %) had problems in more than one area, with a mean of 2.4 defined problem areas (SD = 1.1, median = 2, range 1–6). The mean number of problem areas was significantly higher in females (3.0, SD = 1.3) than in males (2.4, SD = 1.1) (*t*-test p = .02). However, in 13 cases (all males) the therapist specified that the inmate had serious problems with aggression and/or violent behaviour.

**Table 2:** The therapists' gender specific assessments of 230 inmates' main problem areas (each patient may bee allocated more than one problem), and the same problem areas in percentages of the total prison population (N = 928).

**PSYCHIATRIC PROBLEM AREA**	**Male patients N = 210**		**Female patients N = 20**		**Total patient population N = 230**		**Total prison population N = 928**
	
	n	%	n	%	n	%	%
**Psychoactive substance use**	109	52	14	70	123	54	13
**Depression**	98	47	12	60	110	48	12
**Personality deviance**	94	45	10	50	104	45	11
Anxiety *	85	41	13	65	98	43	11
**Posttraumatic stress**	38	18	2	10	40	17	4
**Psychosis**	21	10	4	20	25	11	3
**ADHD**	9	4	4	20	13	6	1
**Sexual deviation**	9	4	0	0	9	4	1
Other **	9	4	0	0	9	4	1

* Significant gender difference (Chi square test, 1DF, p = 0.03)

** Obsessive compulsive disorder, Pathological gambling, Eating disorder, Kleptomania, Adjustment disorder

Among inmates receiving psychiatric interventions, 52 % were receiving psychopharmacological treatment as well.

As already stated, the present study did not concern itself with the variety of correctional programs offered to inmates (anger management programs, etc). Even so, it is worth noticing that 20 % of prisoners in psychotherapy also participated in some of these correctional programs.

The therapist estimated that the patient's therapeutic needs were adequately met in 65 % of cases. Needs were partially met in 29 % of cases and inadequately met in 7 % of cases. One PHS did ascribe this to insufficient psychotherapist capacity. However, most shortcomings pertained to the prison setting itself, with a lack of proper milieu therapeutic facilities and specialised treatment for infrequent conditions.

As a number of group differences were found between remand prisoners, sentenced inmates, and those on preventive detention, results specific to these groups are provided in Table 3.

**Table 3:** Demographic and therapy factors for 230 inmates receiving interventions, according to prisoner category^1^

	**Remand prisoners N = 42**	**Sentenced N = 172**	**On preventive detention N = 16**	**Total therapy population N= 230**
**Male gender **(%)	38 (91)	157 (91)	15 (94)	210 (91)
Mean age *** (SD, range)	30.1 (8.5, 18–50)	33.7 (8.7, 16–56)	42.3 (11.0, 24–64)	33.7 (9.2, 16–64)
Ethnic Norwegian ** (%)	25 (60)	109 (63)	16 (100)	150 (65)
**Psychiatric problem areas**(%)				
Psychoactive substance use	21 (50)	94 (55)	8 (50)	123 (53)
Depression	26 (62)	78 (45)	6 (38)	112 (48)
Personality deviance *	14 (33)	79 (46)	11 (69)	104 (45)
Anxiety	22 (52)	72 (42)	4 (25)	98 (43)
Posttraumatic stress *	3 (7)	37 (22)	0 (0)	40 (17)
Psychosis	5 (12)	18 (11)	2 (13)	25 (11)
ADHD ***	2 (5)	11 (6)	0 (0)	13 (6)
Sexual deviance ***	0 (0)	1 (1)	8 (50)	9 (4)
Other ***	3 (7)	8 (5)	0 (0)	11 (5)
In individual therapy *** (%)	42 (100)	171 (100)	10 (63)	223 (97)
Regular	34 (81)	140 (82)	9 (90)	183 (82)
Ad hoc/emergency session	8 (19)	31 (18)	1 (10)	40 (18)
In group therapy *** (%)	0 (0)	4 (2)	8 (50)	12 (5)
**Psychopharmacological treatment **(%)	26 (63)	87 (51)	6 (38)	119 (52)
**Therapy needs met **(%)				
Yes	28 (74)	102 (61)	13 (87)	143 (65)
Partly	9 (24)	53 (32)	1 (7)	63 (29)
No	1 (3)	13 (8)	1 (7)	15 (7)

^1 ^As all questions had not been answered for all patients, numbers do not always add up to 230 Significant group differences (t-test, Chi square, Exact test): * p ≤ .05, ** p ≤ .01, *** p ≤ 0.001

### The number of consultations provided

Among the 265 remand prisoners 34 (13 %) received planned psychotherapy: 21 had sessions once a week (or more) and 13, 1–3 times a month, all in the form of individual therapies. The total activity amounted to 28 planned therapy sessions per week, or 11 therapy sessions per week per 100 remand prisoners. In addition, 8 inmates (3 %) received ad hoc or emergency interventions during the registration week, i.e. 3 ad hoc therapy sessions per week per 100. The sum of planned and ad hoc individual therapy was 14 sessions per week per 100 remand prisoners.

Among the 602 sentenced inmates, a total of 140 (23 %) received planned psychiatric intervention: 52 had sessions once a week (or more), 85 had sessions 1–3 times a month, and 5 more infrequently. The total activity amounted to 98 planned therapy sessions per week, or 16 sessions per week per 100 inmates. In addition, 31 inmates (5 %) had ad hoc or emergency interventions during the registration week, i.e. 5 sessions per week per 100 inmates. Four (1 %) were in group therapy; this equals 1 session per week per 100 inmates. The sum of planned and ad hoc individual and group therapeutic sessions was 22 per week per 100 sentenced inmates.

Among the 61 on preventive detention, 9 (15 %) were in individual psychotherapy: 3 had sessions once a week and 6 had sessions 1–3 times a month. The total activity amounted to an average of 6 therapy sessions per week, or 10 sessions per week per 100 inmates. In addition, 1 inmate received an ad hoc intervention during the registration week. This amounts to 2 ad hoc therapy sessions per week per 100 inmates. Group therapy was relatively prevalent among those on preventive detention: 8 (13 %) had weekly group therapy. This equals 13 sessions per week per 100 inmates. All in all, prisoners on preventive detention received psychotherapy, either individually or in a group, equivalent to 25 sessions per week per 100 inmates.

## Discussion

In this study we have described in detail all non-pharmacological interventions provided by the PHS in six medium-to-high security prisons. The crucial point is: to what extent did these activities cover the therapeutic needs of the prison population adequately? The number of psychiatric interventions required in any given population is of course highly dependent on the therapeutic goals defined. For instance, dealing adequately with the high number of personality disordered inmates regularly found in any prison would require therapeutic efforts way beyond the capacity of most PHSs. Moreover, a number of therapists would point to the futility of such efforts. We believe that the six PHSs participating in this study had a fairly realistic and balanced view on the potential benefits that can be obtained by psychiatric interventions.

We found that the number of sessions used by sentenced prisoners and prisoners on preventive detention were about the same: 22 and 25 sessions per week per 100 inmates, respectively. Remand prisoners used the PHS less than other inmates (14 sessions per week per 100 inmates), in spite of research showing that they have mental disorders at least to the same degree as sentenced inmates [1,5,12,23]. There might be a number of reasons for this finding. As remand prisoners often stay in prison for a relatively short time their needs may not yet have been detected, or there might not have been sufficient time for the primary health services to complete referral to the PHS. Moreover, psychopathology that arises due to the stressfulness of the incarceration and the impending court case [5] may not yet have developed fully.

Prisoners on preventive detention had more of their needs fulfilled by group therapy. Groups may be easier to conduct for inmates on preventive detention, as they are typically incarcerated for longer periods and less frequently moved between institutions. In addition, they have a higher prevalence of personality problems and sexual deviance, i.e. problems that may be well suited for group therapy [24].

How does the prison population's use of psychiatric services compare with that of the rest of the population? In 2004 the general psychiatric health services delivered 756,900 outpatient consultations to Norway's approximately 3,423,000 inhabitants above 18 years of age [25]. In addition, a number of psychotherapists in private practice [25] delivered at least 584,700 consultations. Altogether, this amounts to 1,341,600 consultations per year or somewhat less than 1 consultation per week per 100 individuals above 18 years of age. Thus, the prison population had a marked increase in consultations, compared with the general population. As already stated prison inmates have higher psychiatric morbidity than the rest of the population. Even so, a 20-fold increase compared to the general population, is substantial.

People with foreign citizenship are over-represented in the forensic system in Norway [26] and represent a particular challenge for the PHS. Special skills are needed in order to conduct therapeutic work via a translator [27]. In addition, the fact that these inmates are alienated from land, culture, family, and language creates special needs that should be addressed in comprehensive rehabilitation programs.

As many as 52 % of those receiving psychiatric consultations did receive psychopharmacological treatment as well, testifying to the widespread use of psychotropic prescription drugs in Norwegian prisons [28].

In asking the therapists to identify their patients' psychiatric problem areas, we did not attempt to arrive at formal diagnoses. Even so, it is of interest to compare the results presented in Tables 2 and 3 with other studies presenting results on the prevalence of psychiatric disorders in prison inmates. Of course the PHS deals with only a fraction of all psychiatric problems in a prison. Most of the less serious problems are adequately addressed by the prison's primary health services; on the other hand most inmates with serious mental disorders should be referred to the PHS. With these reservations, we found that the present study corresponded well with available prevalence studies conducted in the Norwegian prison population [29-31].

Three percent of all inmates in the total prison population were seen by the PHS because they had problems of a psychotic nature, a finding corroborated by a number of studies that have found the prevalence of psychotic disorders in prison inmates to be 2–4 % [1]. However, why is the percentage not zero, when the policy of Norwegian health and prison authorities is that psychotic patients should not be incarcerated? There are a number of prisoners with psychotic disorder who are adequately treated on voluntary anti-psychotic and/or mood stabilising medication. Some of these have been discharged from the psychiatric hospital with a promise of rapid re-hospitalisation if their condition should deteriorate. In addition, some may have, or be recovering from, brief, often drug-induced, psychosis.

The therapists in the study underlined the importance of good collaboration across professions and levels of care. Psychiatric health work with prison inmates should be multidisciplinary, as many diverse perspectives need to be integrated in the care of these patients. Psychiatric nurses play an important role, both at the primary and at the specialist level. In addition, the role of correctional officers should not be underestimated [32]. The optimal climate for effective treatment is one in which mental health professionals and correctional officers work collaboratively [33].

National surveys conducted in the US have demonstrated that the substantial growth in the prisoner population from 1988 to 2000 (114.5 %) has outstripped the more meagre growth in mental health services over the same period. This suggests that mental health services have become less available to the prison population [34]. A study from England and Wales described serious shortcomings and observed that "very few prisons have health care services that reach out to the main prisons, where most of the prisoners with mental health problems and unmet needs are located" [14].

Thus, we have reason to believe that the PHSs in Norwegian prisons have more resources than a number of other countries. Our results do pertain to properly staffed and run prisons, with adequate primary and mental health services and a psychiatric hospital system that is willing and able to service the prison population adequately. Even so, the six participating PHSs differed in their assessments: five testified to the adequacy of the services provided and ascribed any unmet needs to factors pertaining to the prison setting as such, while one PHS assessed the situation as inadequate with insufficient therapist capacity.

### Limitations of the present study

The main weakness of the present study is that it contains no absolute measures of needs and treatment effects; it depends solely on the PHS' opinion of whether or not needs have been met. If we had asked the inmates themselves, the prisons' primary health services, or the prison officers, they might not have agreed. In all research the preferred level of analysis is the one that minimizes confounding. The inmates themselves could have been a natural choice. But even on the person level there may be pitfalls [35]. An individual may, for various reasons, exaggerate his or her needs, or be unduly dissatisfied. Thus, even the person level may not always represent "the gold standard". Ideally, we should have been able to measure objectively the effectiveness of the services provided. This was beyond the scope of the present study. However, even with the weaknesses mentioned, we believe our results merit attention. Future research should include information from multiple levels.

How trustworthy are the mental health workers' reports? Would they not be liable to report favourably on the adequacy and effectiveness of the services they provide, regardless of the actual circumstances? We have reason to believe that this is not the case. There is a long and strong tradition for Norwegian health workers to side with their patients and make public outcry if they consider that health services are inadequately funded or understaffed. Usually, health workers become their patients' advocates and criticise whenever they find this justified. Indeed, a health worker would jeopardise his professional esteem if he signalled that certain services were satisfactory if they in fact were not.

We do not have any measure of the prevalence of mental disorders in the prison population. The study design enables us only to consider the therapy needs of inmates with disorders brought to the attention of and acknowledged by the PHS therapists.

All psychiatric activity may not have been recorded. As stated, primary health service activities were not included. In addition, the inmate's psychiatric needs were not fully met in a minority of cases. Although most of these shortcomings were inherent to the prison setting and, except in one institution, not due to a lack of therapists, the therapy needs estimates must be regarded as minimum requirements.

Due to the selection procedure our findings pertain to medium/large prisons only.

As previously stated, correctional programs are not included in our study. Although the stated goal of these programs is to reduce recidivism, they are obviously important in reaching more general therapeutic goals, such as alleviating suffering and enhancing quality of life.

## Conclusion

In this naturalistic study of mental health services in a prison population, 25 % of the inmates received psychiatric interventions, with14 to 25 psychiatric consultations per week per 100 inmates, depending on prisoner category. The results pertain only to prisons with adequate primary health services and effective diversion from the correctional system of individuals with serious mental disorders. Given these limitations, we do propose that our estimates may serve as a rough guideline to the minimum number of sessions a prison's PHS should be able to fulfil in order to serve the inmates' psychiatric needs. The results rely on the specialist services' own estimates only. Future studies should take other important informants, including the inmates themselves, into consideration.

## Competing interests

The author(s) declare that they have no competing interests.

## Authors' contributions

E. Kjelsberg and P. Hartvig developed the research protocol. All authors participated in developing the questionnaires. Authors 3–8 were responsible for data collection at the participating sites. E. Kjelsberg performed all statistical analyses and drafted the first version of the manuscript. All authors read, gave feedback, and approved the final manuscript.

## Pre-publication history

The pre-publication history for this paper can be accessed here:


